# Infestation risk of the intermediate snail host of *Schistosoma japonicum* in the Yangtze River Basin: improved results by spatial reassessment and a random forest approach

**DOI:** 10.1186/s40249-021-00852-1

**Published:** 2021-05-20

**Authors:** Jin-Xin Zheng, Shang Xia, Shan Lv, Yi Zhang, Robert Bergquist, Xiao-Nong Zhou

**Affiliations:** 1 National Institute of Parasitic Diseases, Chinese Center for Disease Control and Prevention; Chinese Center for Tropical Diseases Research; WHO Collaborating Centre for Tropical Diseases; National Center for International Research on Tropical Diseases, Ministry of Science and Technology; NHC Key Laboratory of Parasite and Vector Biology, Shanghai, 200025 China; 2 School of Global Health, Chinese Center for Tropical Diseases Research, Shanghai Jiao Tong University School of Medicine; One Health Center, The University of Edinburgh, Shanghai Jiao Tong University, Shanghai, 200025 China; 3 Ingerod, Brastad, Sweden/formerly with the UNICEF/UNDP/World Bank/WHO Special Programme for Research and Training in Tropical Diseases (TDR), World Health Organization, Geneva, Switzerland

**Keywords:** Schistosomiasis, *Oncomelania hupensis*, Snail infestation, Yangtze River, Random forest, Spatial sampling, Machine learning, China

## Abstract

**Background:**

*Oncomelania hupensis* is only intermediate snail host of *Schistosoma japonicum*, and distribution of *O. hupensis* is an important indicator for the surveillance of schistosomiasis. This study explored the feasibility of a random forest algorithm weighted by spatial distance for risk prediction of schistosomiasis distribution in the Yangtze River Basin in China, with the aim to produce an improved precision reference for the national schistosomiasis control programme by reducing the number of snail survey sites without losing predictive accuracy.

**Methods:**

The snail presence and absence records were collected from Anhui, Hunan, Hubei, Jiangxi and Jiangsu provinces in 2018. A machine learning of random forest algorithm based on a set of environmental and climatic variables was developed to predict the breeding sites of the *O. hupensis* intermediated snail host of *S. japonicum*. Different spatial sizes of a hexagonal grid system were compared to estimate the need for required snail sampling sites. The predictive accuracy related to geographic distances between snail sampling sites was estimated by calculating Kappa and the area under the curve (AUC).

**Results:**

The highest accuracy (AUC = 0.889 and Kappa = 0.618) was achieved at the 5 km distance weight. The five factors with the strongest correlation to *O. hupensis* infestation probability were: (1) distance to lake (48.9%), (2) distance to river (36.6%), (3) isothermality (29.5%), (4) mean daily difference in temperature (28.1%), and (5) altitude (26.0%). The risk map showed that areas characterized by snail infestation were mainly located along the Yangtze River, with the highest probability in the dividing, slow-flowing river arms in the middle and lower reaches of the Yangtze River in Anhui, followed by areas near the shores of China’s two main lakes, the Dongting Lake in Hunan and Hubei and the Poyang Lake in Jiangxi.

**Conclusions:**

Applying the machine learning of random forest algorithm made it feasible to precisely predict snail infestation probability, an approach that could improve the sensitivity of the Chinese schistosome surveillance system. Redesign of the snail surveillance system by spatial bias correction of *O. hupensis* infestation in the Yangtze River Basin to reduce the number of sites required to investigate from 2369 to 1747.

**Graphical abstract:**

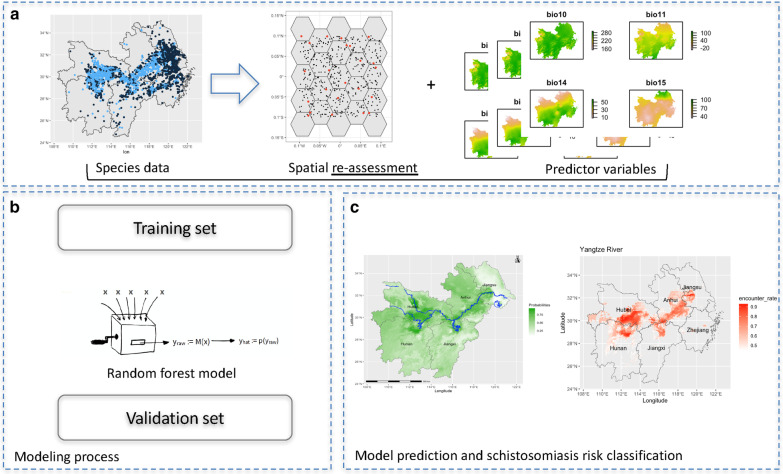

**Supplementary Information:**

The online version contains supplementary material available at 10.1186/s40249-021-00852-1.

## Background

Schistosomiasis, a parasitic serious disease caused by trematode worms belonging to the genus *Schistosoma*, is not only harmful to health but also holds up development of economy and society in the endemic areas [1]. According to the World Health Organization (WHO), the disease is prevalent in 78 tropical and subtropical countries around the world, where it affects the lives of more than 700 million people in the endemic areas, with at least 240 million actually infected. Schistosomiasis thus remains a major public health hazard in the developing world. In contrast to Latin America and Africa, the species in China, *Schistosoma japonicum*, is zoonotic with more than 40 species of mammals as reservoir hosts. The freshwater, amphibious snail *Oncomelania hupensis* serves as the parasite’s intermediate host [2]. Schistosomiasis japonica, once endemic in 12 provinces in southern China [3], is closely associated with the distribution of this snail, which is mainly found in the marshlands of the Yangtze River Basin and connected lake areas [3, 4]. After seven decades of continuous efforts, mainly based on snail control and treatment with the drug praziquantel, transmission interruption has been achieved in nine out of the 12 previously endemic provinces, the lowest level of prevalence ever obtained [5]. The remaining three provinces are located in the lake region in central China along the middle reaches of the Yangtze River. Due to the complex environment that includes annual flooding of the river basin, the risk of resurgence of schistosomiasis remains a constant threat and a major obstacle to accomplishing transmission interruption in the country.

Since *O. hupensis* is *S. japonicum’*s only intermediate snail host, efforts to eliminate schistosomiasis must inevitable include specific snail control to reduce or abolish the transmission risk [6]. These activities have led to a continuing reduction of the endemic areas from year to year as shown by reports from the national surveillance network in China [7]. Notwithstanding, the snail-ridden areas are still so widely distributed in the lake region’s five provinces, including Anhui, Hunan, Hubei, Jiangxi and Jiangsu provinces, that they account for 94.7% of all known snail habitats in the country [8]. This makes the distribution and abundance of *O. hupensis* an important and sensitive indicator for the surveillance system of the national schistosomiasis control programme [9]. Enhanced technology to monitor *O. hupensis* is strongly warranted as improved snail surveillance would make it possible to accelerate the work and realize the goal of schistosomiasis elimination by 2030 as scheduled by the Chinese government [10].

The distribution of *O. hupensis* is closely related to climate and geography, in particular temperature, rainfall, humidity as well as the presence of water bodies and elevations [11, 12]. Mathematical models supported by remotely sensed information can identify and estimate areas suitable for snail infestation in the marshlands [13], while machine learning based on ecological niche modelling could be an additional adjunct as it has been successfully used to predict the distribution of other species [14, 15]. The theory of ecological niche modelling derives from the principle that the distribution of specific species is closely related to their surrounding ecological environment with thresholds for survival that are unique for each species [16]. Although ecological factors associated with the distribution of different species have already been widely employed to predict the geographical distribution of freshwater snails, the precision and scale of the methodology with regard to snail infestation are still not perfected. Niche modelling has performed well when used to estimate the geographic distribution of a species, while machine learning supported by algorithms based on the random forest (RF) approach and decision trees has made even more precise predictions [17]. In order to build linear and non-linear models between species distribution and ecological factors, RF improves classification accuracy and can thus more effectively deal with highly complex data without loss of dimensionality and covariant variables [18]. This approach is highly suitable for the study of the transmission risk posed by schistosomiasis, as the disease is closely related to the distribution of its snail host, which in turn is strongly connected with geography and meteorology, in particular with altering temperatures [12]. The potential distribution of schistosomiasis can in fact be predicted by the surveys using the simple, dichotomous variable ‘Presence/Absence’ of snails at potential breeding sites.

In China, the *O. hupensis* distribution is continuously assessed by the national schistosomiasis surveillance system that records snail presence and relevant ecological data based on sampling [10]. Differentiation between environmentally similar locations are generally not made, and a spatial bias might occur when recorded data are fed into the ecological niche model [19]. Indeed, previous studies on the distribution of *O. hupensis* snails using this approach have seldom accounted for this problem by providing specific, environmental information; only giving the geographical coordinates of the location where the snails were found [20]. In addition, sampling is commonly done in previously surveyed locations, often chosen due to easy reach, while it is highly probable that the true distribution of a species is not limited to locations where it has been observed before [21]. Predictions based on incomplete grounds must be avoided as they will not provide reliable estimations. Thus, the risk of spatial bias arising from historic sampling data needs to be removed before ecological niche investigations are carried out [22].

The aim of this study was not only to avoid the spatial sampling bias of previous surveys, but also to reduce the number of investigated sites in future surveys without losing the level of predictive accuracy required. With this in mind, we used the snail survey carried out in 2018 in the area as reference, applied ecological niche modelling and a RF machine learning algorithm based on an alternative hexagonal spatial grid system.

## Data and methods

The feasibility of designing a superior snail surveillance system was explored by (1) constructing a database of snail infestation based on geographical information systems (GIS) along with ambient environmental data; (2) application of a RF approach based on the evaluation results; and (3) estimating and mapping the most suitable areas for snail infestation.

### Study site

**Fig. 1:**
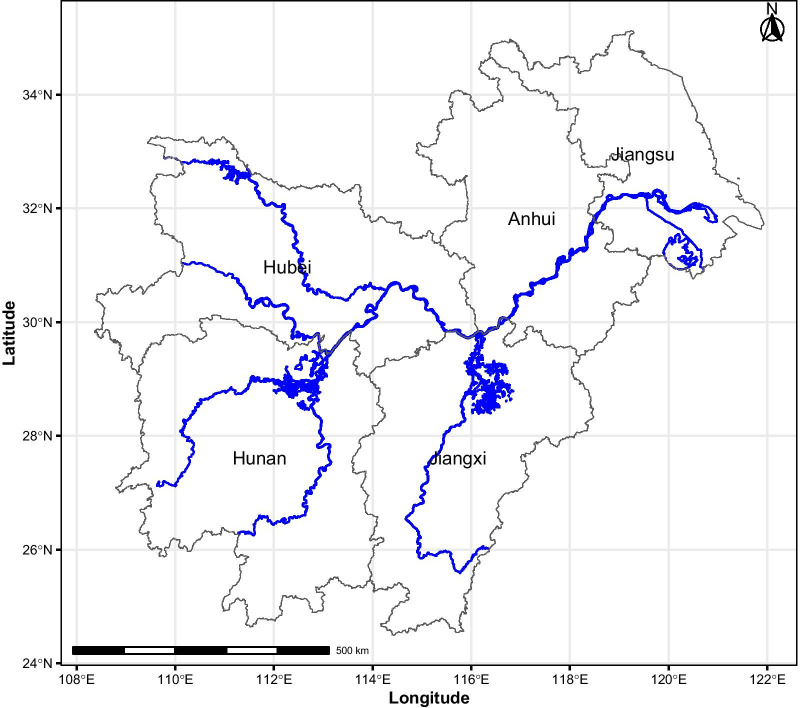
The study area. The Yangtze River Basin is in close contact with the two major lakes, Dongting Lake and Poyang Lake, as it traverses the five endemic provinces in central China

The study was carried out in the Yangtze River Basin (Fig. 1), which constitutes a challenge due to variable geography, long lake shorelines and changing water ways.

### Data source

The starting point was the 2018 data in the national schistosomiasis surveillance system that includes records of all *O. hupensis* snails found in all schistosomiasis-endemic areas with name, location and time for each survey site. Live snail habitats were found in about 50% of the sites investigated. We merged all snail field survey data from the five concerned provinces (Hunan, Hubei, Jiangxi, Anhui and Jiangsu), applied the dichotomous variable ‘Presence or Absence’ of snail infestation and stored this information as raster data in the computer. We also considered the values of a set of environmental variables related to snail infestation and their interquartile ranges (IQRs), a measure based on the difference between the means of the third and the first quartiles that describes data dispersion and variations with non-normal distribution.

**Fig. 2:**
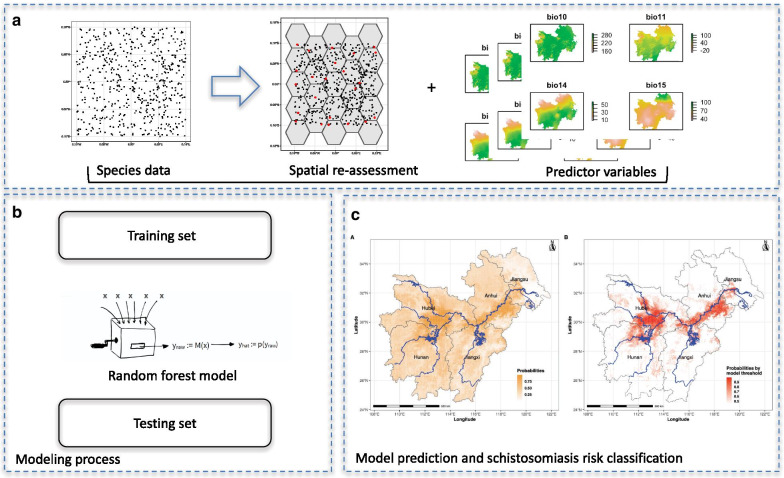
Flow diagram of the study. **a** The snail distribution dataset in the form of a spatial shapefile including ‘presence–absence’ records (left); re-assessment to correct the spatial bias (middle); environmental and geographic predictor variables prepared as raster files of the area (right). **b** Random forest model first fitted with the training set followed by testing by the validation set. **c** Probability of snail occurrence (left) and risk output based on thresholds (right)

The data used included geographical information (altitude, distance from water bodies and land cover), human activity and climate data. The former evidence was provided by the Socioeconomic Data and Applications Center of China (http://www.resdc.cn/), and the meteorological information came from the WorldClim database (https://www.worldclim.org/data/bioclim.html) of 19 biologically significant variables (Table 1). All predictor variables were converted into spatial raster data as shown in Fig. 2.

**Table 1:** Variables used in the study

Variable	Value	Interquartile range (IQR)	Source
Altitude (m)	141.5	32.8–396.4	Socioeconomic Data and Applications Center of China
Human influence index (HII)^a^	20.1	16.9–25.5	
Human foot print^b^	31.1	26.1–39.2	
Land cover	10	4–16	
Distance to the river (km)	9.5	4.7–66.5	
Distance to other water bodies (km)	74.5	28.7–156.3	
Annual mean temperature (℃)	16.1	15.2–17.1	WorldClim BIO1
Mean diurnal range (max – min temp) (℃)	8.1	7.6–8.5	WorldClim BIO2
Isothermality^c^ (%)	25.0	24.1–26.26	WorldClim BIO3^d^
Temperature seasonality	8346.7	7894.1–8718.5	WorldClim BIO4^e^
Max temperature of the warmest month (℃)	32.1	30.9–32.9	WorldClim BIO5
Min temperature of the coldest month (℃)	3.9	-14.1–15.5	WorldClim BIO6
Temperature annual range (℃)	31.7	30.5–32.8	WorldClim BIO7^f^
Mean temperature of the wettest quarter (℃)	22.6	21.2–25.7	WorldClim BIO8
Mean temperature of the driest quarter (℃)	6.4	4.5–8.6	WorldClim BIO9
Mean temperature of the warmest quarter (℃)	26.8	25.8–27.5	WorldClim BIO10
Mean temperature of the coldest quarter (℃)	51.8	37.1–62.5	WorldClim BIO11
Annual precipitation (mm)	1374.8	1053.6–521.1	WorldClim BIO12
Precipitation of the wettest month (mm)	218.5	196.4–245.2	WorldClim BIO13
Precipitation of the driest month (mm)	38.1	27.1–45.1	WorldClim BIO14
Precipitation seasonality (mm)	53.1	48.7–59.0	WorldClim BIO15^g^
Precipitation of the wettest quarter (mm)	584.5	476.2–650.7	WorldClim BIO16
Precipitation of the driest quarter (mm)	139.9	98.8–169.7	WorldClim BIO17
Precipitation of the warmest quarter (mm)	487.8	448.2–544.1	WorldClim BIO18
Precipitation of the coldest quarter (mm)	152.8	100.8–194.6	WorldClim BIO19

^a^measure of direct human influence on terrestrial ecosystems based on (1) human settlement; (2) access, such as roads, railroads, rivers, etc.; (3) land use/land cover; and (4) night-time electric light. Values range from 0 to 64, where the former represents no human influence and the latter maximum influence; ^b^heatmap representation of human power over nature on the land surface, ranging from red (highest) to green where wildness still thrives; ^c^measure of how large the day-to-night temperatures oscillate relative to the summer-to-winter oscillations; ^d^BIO2/BIO7 × 100; ^e^BIO 4 = standard deviation × 100; ^f^BIO5-BIO6; ^g^Coefficient of variation

The human influence index consists of a global dataset created from nine global 1 km^2^ data layers covering human population density, land use/land cover, infrastructure (built areas, night-time light) and access (roads, railroads, navigable rivers, and harbours on coastlines), represented by a 0–64 range corresponding to values from no influence to the currently highest [23]. The human footprint is a heatmap using the factors mentioned to illustrate the degree of human domestication of nature on a scale from high impact to still untouched natural areas that can also be given as numerical values as seen in Table 1.

### Re-assessment of sampling sites

Vector structures are generally based on grids with square lattices. However hexagonal grids, as suggested by Sahr [24], unlike square or circle grids, produce significantly less spatial distortion that all grid cells cover the same area, which is particularly important when applied for large regions. There are severe resource constraints for efficient processing of raster and vector data in high-performance applications. We discovered the hexagonal discrete global grid system (HDGGS) [24], when searching for a highly efficient approach for location representation that would reduce the need for snail sampling sites while randomizing the records to be evaluated. Decomposing the study area into a set of adjacent hexagonal cells provided us with a surface unit that not only fits the eye’s retinal reception better than a square but also is much closer to a circle where the distances to the periphery are more similar than in a square. If the distance between locations in two different cells is smaller than desired, the cell “radius” can be enlarged leading to the study area consisting of a lower number of larger cells. Thus, for each set distance between immediately adjacent cell centres, there is a corresponding number of cells covering the region studied.

**Fig. 3:**
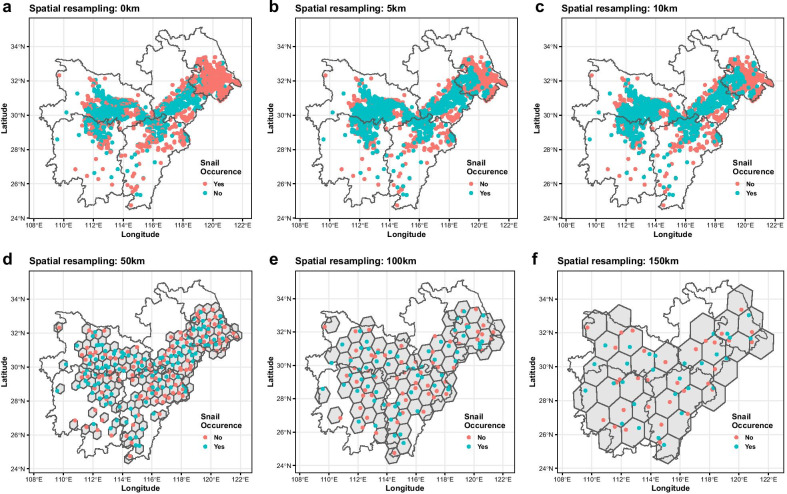
Distribution of the snail records in the Yangtze River Basin with increasing cell sizes. **a** Snail records according to the 2018 field survey; **b**–**f** Spatial correction using 5, 10, 50, 100 and 150 km distance between the centres of adjacent cells, respectively. Snail records (with red for presence and blue for absence) adjusted to keep 1 or two records per grid cell. Note that cells are not shown for Figures **a**–**c** since the sizes would have been too small at those scales

Considering the spatial bias of snail records when estimating the suitability level of snail infestation in various areas, re-assessed values based on five different cell distance scenarios were investigated using 5, 10, 50, 100 and 150 km between the centres of adjacent grid cells, while 0 km corresponded to the previous survey snail site records. In this way, the sites came to move between adjacent hexagons in a randomized way (Fig. 3). With the cell centre value(s) representing the whole cell, unnecessary sampling sites could be bypassed (erased in the figure) to make each hexagon size in principle contain only one sampling site (the one closest to the centre).

### Model construction

The desired balance between accurate prediction and simple assumption plays an important role in ecosystem assessment, and the distribution of snail colonies requires highly accurate predictions of spatial patterns. The RF machine learning algorithm is useful as it builds multiple decision trees and merges them to get accurate and stable predictions [25]. As it is both flexible, accurate, and highly correlated, variables won’t cause multi-collinearity issues in RF [26], then this algorithm was selected for various simulations of the ecological niche model to generate predictions regarding the presence of snail habitats.

The re-assessed snail records were incorporated into the model with the dichotomous variable ‘Presence or Absence’ of snail infestation sites as the dependent variable, and with the 25 ecological environmental factors shown in Table 1 as predictor variables. When complete, the dataset was split into a training set (80%) and a validation set (20%). To prevent statistical overfitting, a cross-validation approach was used to minimize observation error of screening only appropriate RF nodes in the training set. We also used calibration to diagnose the predictions by the training, which generated 100 segment intervals between 0 and 1, then grouped the snail infestation probability and observations in each segment interval to calculate the average predicted and observed rates [27]. A linear model was used to analyse the correlation between prediction and observation. After the calibration, we used this RF model to assess the predictive performance in the validation set. The prediction accuracy of the model was evaluated by the receiver operating characteristic (ROC) approach calculating the area under the curve (AUC) [28], as well as by the Kappa value [29]. AUC varies between 0 and 1 and the larger the value, the better the predictive accuracy, while the Kappa value varies between 1 and -1 and the closer it is to 1, the better the model prediction consistency and reliability, while negative values indicate random outcomes.

The RF was established to predict the snail probability in the training set, then followed by testing using the validation set. This decision tree approach computes the reduction sum of the loss functions across all splits, then aggregate this measure across all trees for each feature and the one with the largest average decrease is considered the most important [25]. RF variable importance measures are a sensible means for variable contributions for the purpose of response variables in classification, and shows the most important variable from noisy variables [30]. By randomly permuting each predictor variables, and its original association with the purpose of responses [31], a normal calculation method in machine learning, it is possible to find indicators to which predictors are particularly important to the model. Through the RF model, the partial dependence of the marginal effect of each variable in relation to *Oncomelania* snail infestation probability can be calculated with the result describing the interrelationship between snail infestation probability and each predictor variable [32]. Considering the numbers of variables and permutations, the partial dependence only the importance of variables exceeding 25% from the best fit model. In this study, we explored this by finding out which variables performed best after re-assessing the sampling sites.

### Areal suitability for snail infestation

We used the RF algorithm to predict the range of varying degrees of suitability for snail infestation in the study area based on the ecological factors given in Table 1 and expressed the results in a ‘heatmap’, where each point shows the level of suitability for snail infestation on a range between 0 and 1. It was necessary to divide the snail infestation areas by threshold levels, as they indicated the probabilities of snail presence that could be translated into predicted probabilities by the cut-off value, which is a positive integer representing the number of evenly spaced thresholds. The default criterion of setting the threshold value is 0.5, and in our study we choose thresholds that maximized the sum of sensitivity and specificity and minimized the mean of the error rate for positive observations and the error rate for negative observation [15]. Considering the ecological characteristics of the *Oncomelania* snail, the use of the temperature gap for snail breeding between 18 °C and 28 °C was seen as highly important as it produced cut-off points for the prediction of snail infestation suitability [33].

### Data analysis

The statistical analysis and mapping used in this study were performed in R software (Vienna, Austria, version 4.0.2) The main computational packages included the Discrete Global Grids for R package dggridR (https://rdrr.io/cran/dggridR/f/vignettes/dggridR.Rmd) that was used for the spatial re-sampling approach; the raster package (https://rspatial.org/raster/raster/RasterPackage.pdf) for processing the raster data in snail survey and predictor variables; the Ranger package (https://cran.r-project.org/web/packages/ranger) for constructing RF models, also with variable importance permutations, marginal effect calculations and forecasting, and the R package ggplot2 (https://www.r-graph-gallery.com/ggplot2-package.html) for data visualization.

## Results

### Descriptive analysis

The 2018 survey of snail records from Hunan, Hubei, Jiangxi, Anhui and Jiangsu provinces amounted to 2369 sites, out of which 1061 were positive, i.e. live snails were found, giving a ratio of 0.448. Figure 3a depicts the geographical distribution of these survey sites over the Yangtze River Basin. Table 1 shows the extracted values from the raster map in relation to the various predictive variables and the calculated IQRs.

### Calculation of the most suitable re-assessment level

The number of hexagonal grid cells decreases with increasing distance between the centres of adjacent grids (Additional file 1: Table S1) leading to a lower number of geographic locations of snail records needed per cell as seen in Fig. 3. As the value at the center of each cell will represent the value of the whole cell, we only needed these pilot points for the evaluation of the whole area, which means that the number of re-assessed sample sites is equal to the number of grid cells.

**Fig. 4:**
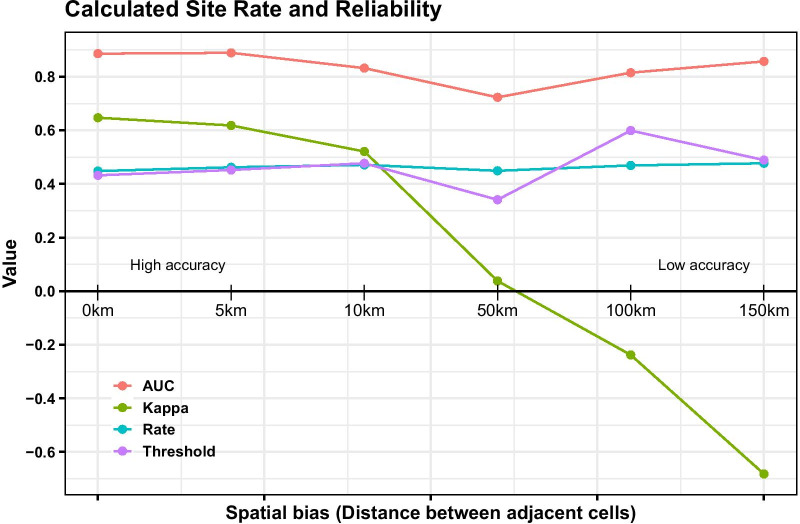
Plot of the random forest model on validation set by different spatial resampling of snail records. Distance refers to the gap between the centres of adjacent grid cells; Rate refers to the ratio of the number of recorded snail infestation sites to that of the total investigated sites. Threshold, Kappa and AUC were determined by random forest model validation set

The performance of the validation set is depicted in the Additional file 1: Table S1. The overall ratio of positive snail infestations to the total number of sites per area increased from 0.448 (the whole study area) to 0.477 (the largest cell tested) even if the value varied from each cell enlargement to the next (Fig. 4 and Additional file 1: Table S1). However, the Kappa values decreased rapidly towards total random values with increasing cell sizes, while the AUC remained relatively stable though ending up slightly below the initial value (Fig. 4). We aimed to find the lowest number of survey sites without losing surveillance resolution (sensitivity and specificity) as the distances grew from 0 to 150 km and the number of hexagonal cells decreased (Fig. 3b–f). This was achieved at the 5 km distance weight that produced 1747 sample sites with the highest accuracy (AUC = 0.889; Kappa = 0.618).

### Variable importance and model calibration

**Fig. 5:**
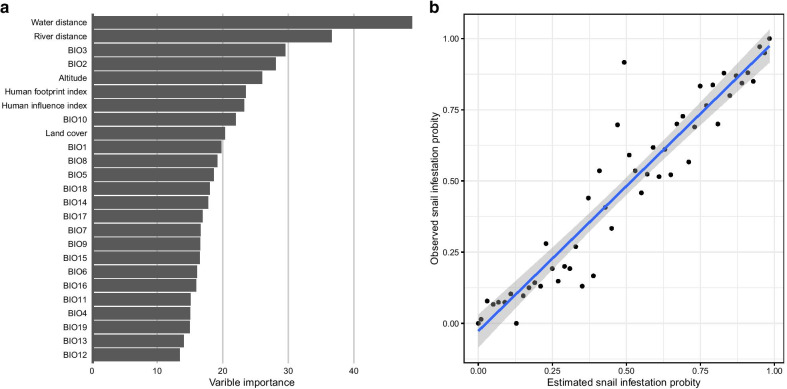
Variable importance and model calibration of random forest at 5 km spatial re-assessment. **a** The ranks of contribution by all variables to the occurrence probability of snail infestation. **b** Calibration of model prediction in the training set, with a good fitness with the real observed snail infestation probability as the response and the predicted snail infestation probability as the predictor variable

With the RF established, the highest AUC (the one giving the best prediction of snail infestation for the study area) was found to be at the 5 km distance, which was therefore chosen for further analysis. The variable importance in the stochastic model established was ranked according to contribution to the environmental suitability for snail infestation (Fig. 5a). The five variables with importance values exceeding 25% were ranked in falling importance as follows: (1) distance to lake (48.9%), (2) distance to river (36.6%), (3) isothermality (BIO3) (29.5%), (4) mean daily difference in temperature (BIO2) (28.1%) and (5) altitude (26.0%). The importance of the rest of variables is shown in Additional file 1: Table S2.

Based on these spatially re-assessed data of snail records, the calibration of the RF training set showed that the established RF model had a good fitness for snail occurrence when the observed snail survival probability was either small or large (Fig. 5b). However, when the snail survival probability was between 0.25 and 0.40, the estimated probabilities were much larger than that those observed. Overall, however, a high correlation (R^2^ = 0.89) between estimation and observation was obtained. The calibration of model prediction in the training set showed a good fit with the observed snail infestation sites as the response and the predicted snail infestation probability coincided.

### Relationship between variables and snail infestation probability

**Fig. 6:**
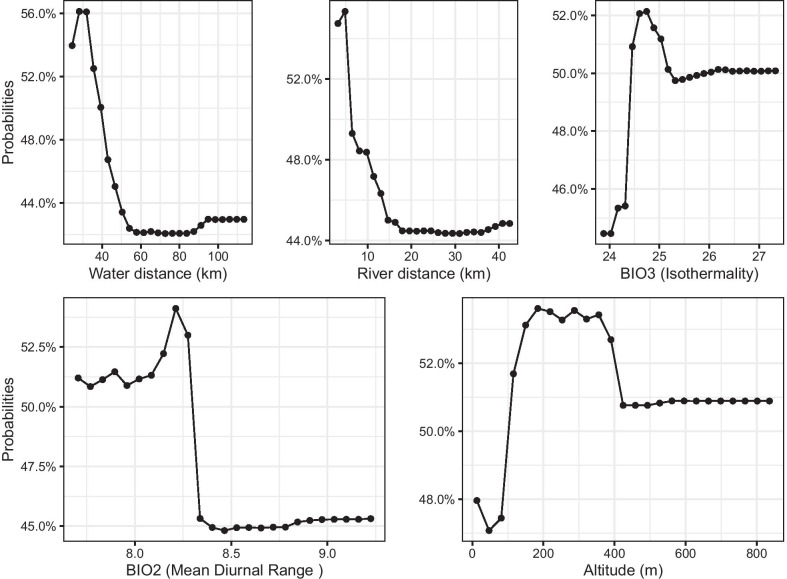
Dependence plots of the top five variables based on random forest model at 5 km spatial re-assessment

Based on the variable importance resulting from the RF, the top five variables correlated well with the snail infestation probability (Fig. 6), but each variable had a different effect on snail infestation and all showed a non-linear relationship. First, the snail infestation probability was the highest when the distance to the water body was below 30 km, but it was still as high as 56%, decreasing to 42% and less further away; Second, with a 5 km distance to the river, the probability reached 55.3% before decreasing with longer distances; Third, the probability of snail infestation increased with isothermality and when this value reached 25.3%, the probability maintained a value around 51%; Fourth, with the mean diurnal range of temperature increased, the impact on snail infestation probability first increased and then decreased. When the mean diurnal range of temperature was 8.2 °C, the probability of snail infestation reached a maximum around 53%; Fifth, as the altitude increased, the probability of snail infestation also first increased and then decreased to a stable level but still reaching 54% at altitudes of 200–400 m.

### Risk prediction

**Fig. 7:**
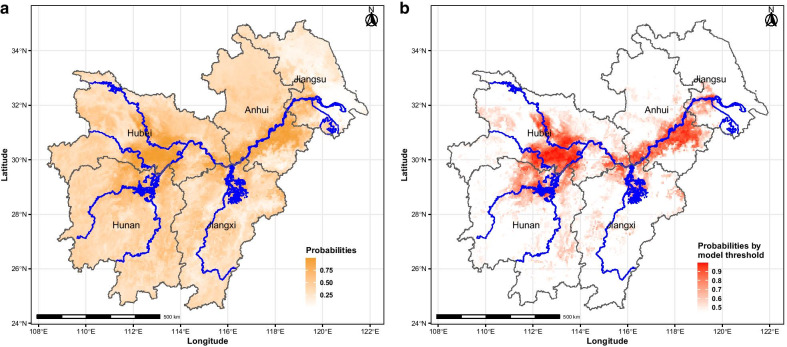
Prediction of schistosomiasis risk based on the 5 km spatial re-assessment in the five provinces of the Yangtze River Basin. Probability of snail infestation by **a** the random forest model, and **b** the threshold cut-off

The predicted risk map was created based on predictor variables of snail infestation probability (Fig. 7a), including model thresholds and the temperature cut-off levels. The risk map showed that the snail infestation probability was higher in areas near or inside the Yangtze River Basin, particularly in Hubei and Anhui provinces (Fig. 7b).

## Discussion

The provinces traversed by the Yangtze River Basin have been able to achieve and maintaining the status of transmission control but large snail-infested areas still exist, forcing snail surveillance to be continued [2, 4]. With the progress of schistosomiasis elimination programme in China, the comparative cost of maintaining snail control in these areas is rising. Only accurate risk predictions of *O. hupensis* infestation and a full understanding of the schistosomiasis transmission patterns in this area can slow this trend.

As the sole intermediate host of *S. japonicum*,* O. hupensis* snail is strictly aligned with endemic areas of schistosomiasis japonica, a fact that not only reflects the importance of knowing its distribution in general but also the degree of infection in the snail population [10]. For the national schistosomiasis surveillance system, it is essential to develop an easily implemented approach to precisely predict these two pieces of information [9]. Since ordinary snail sampling is both labor-intensive and less precise than required, we chose this area and explored the RF machine learning algorithm for snail records aggregated with environmental and climate data to design a new snail surveillance system with fewer snail sampling sites but without losing predictive accuracy. These new approaches were bundled together using a hexagonal base layer as geographical reference because of the environmental variation within the hexagon is less likely to vary compared to the square whose four corners are further away from the center.

The finding of a 5 km between hexagon centres as the most suitable distance, indicates that the number of survey sites needed to precisely predict the real status of snail infestation in the Yangtze River Basin could be decreased from 2369 to 1747 (Additional file 1: Table S1). The advantage of this strategy of designing surveillance sites for routine snail surveys is that it would be less costly, yet more precise. For estimating the predictability of the new approach, AUC and Kappa, both powerful tools for accuracy estimation of ROC analysis, were applied. The former is particularly useful for classifying binary-class problems, such as the snail presence/absence dichotomy, while that latter compensates for random success in multi-class cases. An understanding of how these two accuracy meters relate to each other has been revealed [34], and as it can assist the understanding of their respective advantages we used both methods to make sure to find out at which distance the outcome was sufficiently accurate.

The use of the RF machine learning algorithm to predict the best suitability of snail infestations provided a simple way to perform the re-assessment analysis in the study area. Re-assessing the snail records using hexagon cells with a “radius” of 5 km produced an AUC value of 0.889, which thus indicated a better prediction performance than the use of the original sites (at 0 km distance); indeed, this AUC value was better than any of the larger distances investigated (Additional file 1: Table S1). When the distance between cell centers increased, the predictive capacity, as indicated by the AUC fell reaching a low at the 50 km distance. However, long before this point was reached (Fig. 4), the Kappa value had edged closer to the negative, thus giving a random verdict that indicates that the prediction results cannot be trusted [29]. Although the number of grid cells decreased to 44 at the 150 km hexagon “radius”, this is hardly useful in practice. As indicated by both AUC and Kappa measurements, the lowest useful number of sites (1747) was found at the 5 km level. Hence, it is extremely important to use spatial weighting approach before applying prediction modelling.

Many studies have used ecological niche modelling to predict the probability of the freshwater snail distribution based on the snail records as the response variable, but they have generally ignored the spatial bias invoked as routine snail survey data are not distributed randomly [35, 36]. This spatial bias is due to the uneven distribution of snails in the natural environment, e.g., there are often more than one thousand snail records within a radius of 5 km, but as some sites investigated are closer to each other than others, the result invariably becomes a negative binomial distribution. In addition, when sites investigated are from similar environments with respect to variables, such as altitude, temperature or land cover, they must be considered repeat records and therefore generating redundant data in model processing, which leads to statistical overfitting.

Since *O. hupensis* is an amphibious snail that needs water for reproduction and survival, the greatest factor with regard to snail infestation was unsurprisingly found to be the distance to water bodies, a result consistent with previous observations near the Yangtze River [37, 38]. Second, human activities have a non-linear level of impact on the probability of snail infestation but the footprint shows the lowest values in unperturbed environments where the probability of *O. hupensis* survival consequently is high, in contrast to urban and industrial areas where *O. hupensis* habitats are scarce. Third, the temperature range was found to be extremely important for snail survival as it demands a narrow range (18–28 °C) for replication and development [39], and cannot survive at all if the lowest mean temperature of the year falls below freezing [1]. Even temperature variation plays a role, the snail infestation probability was over 50% when the average daily temperature differences were less than 8.2 °C above which the probability dropped sharply. The larger the difference in daily temperatures, the lower the probability for water or moisture and this threatens *O. hupensis* reproduction, both due to interrupted hatching of its eggs and massive death of the adult snails when areas dry out [40].

The results discussed here show that the areas along the Yangtze River provide the highest suitability for snail infestation, in particular in Hubei and Anhui, where the snail infested areas account for 96.8% of the national total [41]. The former is a typical example of lake region transmission, which is concentrated in the central and southern regions of the province [38, 42], while the risk areas in Anhui province are found along with the river systems [43]. These are the areas of the higher risk for schistosomiasis transmission in the country and further progress towards the elimination of schistosomiasis majorly depend on improved snail surveillance.

As traditional methods for snail surveys are labor-intensive and time consuming, RF supported by machine learning could make a real difference here [7]. Snail control strategies also need to be improved with a focus on focal mollusciciding in high-risk areas with infected snails, which could be further facilitated by similar approaches including remote sensing for the detection of areas suitable for snail habitats [44], particularly during massive flooding along the Yangtze River, as occurred in 1998 and 2020, which created many new snail-infested habitats [33]. Here also, snail infestation probability based on environmental and meteorological factors can be used to improve the sensitivity of the surveillance systems of the national schistosomiasis elimination programme in China [45].

Although we accounted for both environmental factors and human behavioural changes, a limitation was that the latter is not yet as precise as the former. In addition, even if the new WorldClim version of 2017 provides updated data [46], the impact of climate change is now moving so fast that even five years old data are not precise enough for today’s assessment of the risk posed by the *O. hupensis* snails with regard to schistosomiasis transmission [30, 33]. Second, we only used one RF machine learning algorithm to predict the snail infestation potential, and it would be useful to also investigate others as it might be possible to find and select another method providing even better data. Third, our current modelling approach emphasized the snail distribution but did not take into account the role of the degree of infection of the snails, which is equally important. However, this component requires a different approach and was therefore left for future studies.

Since the initiation of the national schistosomiasis control programme was started in China 70 years ago, snail control always had a prominent place [47]. Even if the introduction of praziquantel changed the approach to schistosomiasis control completely, snail surveillance remains of high importance in the programme [48]. Therefore, the national schistosome surveillance systems must be further improved to achieve schistosomiasis elimination in China.

## Conclusions

Data on snail infestations must be provided in real time at a high predictive accuracy, as this information is critical for the national schistosomiasis elimination programme in China. Based on the results of the study, schistosomiasis surveillance should be strengthened with special reference to snail distribution, especially along the Yangtze River. It is expected that the results of this study will improve the design of the national surveillance system.

## Supplementary Information

**Additional file 1: Table S1.** Variation of random forest parameters in relation to spatial distance. **Table S2.** Variable importance of RF model with spatial distance in 5 km.

## Data Availability

The datasets used and/or analysed during the current study are available from the corresponding author on reasonable request.

## Abbreviations

AUC: Area under the curve
GIS: Geographical information systems
HDGGS: Hexagonal discrete global grid system
IQRs: Interquartile ranges
RF: Random forest
ROC: Receiver operating characteristic
