# Supplementary material for: Infestation risk of the intermediate snail host of Schistosoma japonicum in the Yangtze River Basin: improved results by spatial reassessment and a random forest approach
Source: Infect Dis Poverty. 2021 May 20;10:74. doi: 10.1186/s40249-021-00852-1 (PMC8135174; doi:10.1186/s40249-021-00852-1)
Supplement: Supplementary file 1 — Additional file 1: Table S1. Variation of random forest parameters in relation to spatial distance. Table S2. Variable importance of RF model with spatial distance in 5 km. [file 40249_2021_852_MOESM1_ESM.docx]

**Supplementary**

The Table S1. shows the performance of the RF model on validation set by different spatial resampling of snail records. After each spatial resampling, the RF model parameters are estimated, and the Figure 4 output is based on Table S1 as below.

**Table S1. Variation of random forest parameters in relation to spatial distance**

| **Distance^*^** | **No. of cells** | **Rate** | **Ratio** | **Threshold** | **Kappa** | **AUC** |
| --- | --- | --- | --- | --- | --- | --- |
| 0 km | N.A. | 0.448 | 1061/2369 | 0.432 | 0.647 | 0.886 |
| 5 km | 1,747 | 0.462 | 807/1747 | 0.452 | 0.618 | 0.889 |
| 10 km | 1,421 | 0.471 | 669/1421 | 0.477 | 0.521 | 0.832 |
| 50 km | 209 | 0.449 | 93/209 | 0.341 | 0.038 | 0.723 |
| 100 km | 98 | 0.469 | 46/98 | 0.599 | -0.238 | 0.815 |
| 150 km | 44 | 0.477 | 21/44 | 0.489 | -0.682 | 0.857 |

Note: Distance refers to the gap between the centres of adjacent grid cells; Rate refers to the Ratio of the number of recorded snail infestation sites to that of the total investigated sites; Threshold, Kappa and AUC (area under the curve) were determined by random forest model validation set.

**Table S2. Variable importance of RF model with spatial distance in 5km**

| Variables | Variable importance (%) |
| --- | --- |
| Water distance | 48.90 |
| River distance | 36.63 |
| BIO3 | 29.54 |
| BIO2 | 28.11 |
| Altitude | 26.02 |
| Human footprint index | 23.49 |
| Human influence index | 23.25 |
| BIO10 | 21.99 |
| Land cover | 20.32 |
| BIO1 | 19.8 |
| BIO8 | 19.2 |
| BIO5 | 18.62 |
| BIO18 | 18.00 |
| BIO14 | 17.77 |
| BIO17 | 16.91 |
| BIO7 | 16.64 |
| BIO9 | 16.52 |
| BIO15 | 16.49 |
| BIO6 | 16.06 |
| BIO16 | 15.96 |
| BIO11 | 15.10 |
| BIO4 | 15.00 |
| BIO19 | 14.95 |
| BIO13 | 14.02 |
| BIO12 | 13.41 |

Note: The mean decrease in impurity (Gini) importance metric describes the improvement in the “Gini gain” splitting criterion (for classification only), which incorporates a weighted mean of the individual trees’ improvement in the splitting criterion produced by each variable.
